# Temporal transformer-spatial graph convolutional network: an intelligent classification model for anti N-methyl-D-aspartate receptor encephalitis based on electroencephalogram signal

**DOI:** 10.3389/fnins.2023.1223077

**Published:** 2023-08-28

**Authors:** Ruochen Dang, Tao Yu, Bingliang Hu, Yuqi Wang, Zhibin Pan, Rong Luo, Quan Wang

**Affiliations:** ^1^Key Laboratory of Spectral Imaging Technology, Xi’an Institute of Optics and Precision Mechanics (XIOPM), Chinese Academy of Sciences, Xi’an, China; ^2^School of Electronic and Information Engineering, Xi’an Jiaotong University, Xi’an, China; ^3^University of Chinese Academy of Sciences, Beijing, China; ^4^Key Laboratory of Biomedical Spectroscopy of Xi’an, Xi’an Institute of Optics and Precision Mechanics (XIOPM), Chinese Academy of Sciences, Xi’an, China; ^5^Department of Pediatrics, West China Second University Hospital, Sichuan University, Chengdu, China; ^6^Key Laboratory of Obstetric and Gynecologic and Pediatric Diseases and Birth Defects of Ministry of Education, Sichuan University, Chengdu, China

**Keywords:** anti NMDA receptor encephalitis, viral encephalitis, EEG, transformer, graph network, classification

## Abstract

Encephalitis is a disease typically caused by viral infections or autoimmunity. The most common type of autoimmune encephalitis is anti-N-methyl-D-aspartate receptor (NMDAR) antibody-mediated, known as anti-NMDA receptor encephalitis, which is a rare disease. Specific EEG patterns, including “extreme delta brush” (EDB), have been reported in patients with anti-NMDA receptor encephalitis. The aim of this study was to develop an intelligent diagnostic model for encephalitis based on EEG signals. A total of 131 Participants were selected based on reasonable inclusion criteria and divided into three groups: health control (35 participants), viral encephalitis (58 participants), and anti NMDAR receptor encephalitis (55 participants). Due to the low prevalence of anti-NMDAR receptor encephalitis, it took several years to collect participants’ EEG signals while they were in an awake state. EEG signals were collected and analyzed following the international 10–20 system layout. We proposed a model called Temporal Transformer-Spatial Graph Convolutional Network (TT-SGCN), which consists of a Preprocess Module, a Temporal Transformer Module (TTM), and a Spatial Graph Convolutional Module (SGCM). The raw EEG signal was preprocessed according to traditional procedures, including filtering, averaging, and Independent Component Analysis (ICA) method. The EEG signal was then segmented and transformed using short-time Fourier transform (STFT) to produce concatenated power density (CPD) maps, which served as inputs for the proposed model. TTM extracted the time-frequency features of each channel, and SGCM fused these features using graph convolutional methods based on the location of electrodes. The model was evaluated in two experiments: classification of the three groups and pairwise classification among the three groups. The model was trained using two stages and achieved the performance, with an accuracy of 82.23%, recall of 80.75%, precision of 82.51%, and F1 score of 81.23% in the classification of the three groups. The proposed model has the potential to become an intelligent auxiliary diagnostic tool for encephalitis.

## 1. Introduction

Encephalitis is typically caused by viral infections or autoimmunity. The most frequent cause of autoimmune encephalitis is the presence of anti-N-methyl-D-aspartate receptor (NMDAR) antibodies, which is commonly referred to as anti NMDA receptor encephalitis (Nosadini et al., 2021). N-methyl-D-aspartate receptors (NMDARs) are a specific type of ionotropic glutamate receptors that are widely distributed in the central nervous system. These receptors play a crucial role in synaptic plasticity and a wide range of cognitive functions (Adell, 2020). However, the presence of anti NMDAR antibodies can interfere with their function by cross-linking and internalizing them into neurons, leading to a functional deficiency of NMDARs (Hughes et al., 2010). Anti NMDA receptor encephalitis is a rare condition, classified as ORPHA:217253, and its clinical diagnosis is based on the presence of characteristic symptoms and the detection of autoantibodies in the cerebrospinal fluid and serum (Graus et al., 2010).

Electroencephalogram (EEG) is widely used in the examination of patients with anti NMDA receptor encephalitis, and the abnormal rate is beyond 80%. Various EEG patterns have been reported, including diffuse or focal slow wave intermittent or continuous discharges, epileptiform discharges, and others (Gillinder et al., 2019). EEG features have been identified to be useful in diagnosis and prognosis in anti NMDA receptor encephalitis (Freund and Ritzl, 2019). Adequate analysis was conducted on EEG data from 62 patients in China who had anti NMDA receptor encephalitis, revealing characteristic electroencephalogram abnormalities. The majority of these patients showed abnormal EEG signals, including the common diffuse slowing presentation (Zhang et al., 2017). Encephalitis can have various causes, making it crucial to identify infectious etiologies. In a previous study, the comparison between anti NMDA receptor encephalitis and viral encephalitis was examined. The monitoring of EEG signals in patients with anti NMDA receptor encephalitis demonstrated a universal diffuse slowing feature and less focal epileptic activity when compared to viral encephalitis. Moreover, abnormal EEG signals in the temporal lobe may indicate a viral etiology (Gable et al., 2009). Additionally, a highly specific pattern called “extreme delta brush” (EDB), characterized by rhythmic bursts of slow-wave and superimposed fast-wave activity across the delta, has been found in up to one-third of patients (Schmitt et al., 2012). Another study comparing pediatric and adult patients with anti NMDA receptor encephalitis noted that 50% of adults and 33% of children exhibited EDB (Huang et al., 2016). EEG data from pediatric patients during the acute stage of the disease have revealed diffuse alpha-theta rhythms of high amplitude, mainly in the anterior region, with reduced normal slow wave activity during sleep, which is similar to the EEG pattern recorded in the awake state (Gitiaux et al., 2013). Based on these related studies, it is suggested that EEG features could potentially function as a biomarker for the detection of encephalitis. With advances in computational analysis techniques and machine learning techniques (ML), the clinical application of EEG signatures may be significantly improved.

The transformer block is a structure based on the attention mechanism, which has been widely used in image processing and has demonstrated excellent performance in image processing tasks (Dosovitskiy et al., 2010). Song et al. (2022) introduced the convolutional transformer model, which utilizes convolutional modules to learn local temporal and spatial features and self-attention modules to learn global temporal features. The model extracted key information from EEG data on a global level and achieved good performance on three public datasets. Previous studies have used transformer blocks in spatial and temporal ways. In the spatial-wise approach, the transformer block computes the correlations among individual EEG channels, whereas in the temporal-wise approach, it calculates the correlation between different time points (Xie et al., 2022). Li et al. (2022) proposed a hybrid network that combined Convolutional Neural Networks (CNN) and transformer to extract local and global information from STFT-transformed images. This structure compensated for the weaknesses of CNN and transformer by using short-time Fourier transform (STFT) to extract time–frequency features from EEG signals. To address the issue of EEG emotion recognition, Liu et al. (2022) built the EEG emotion transformer (EeT) framework using several variants of self-attention blocks, including spatial (S) attention, temporal (T) attention, sequential spatial–temporal (S-T) attention, and simultaneous spatial–temporal (S + T) attention. The results showed that the simultaneous spatial–temporal attention achieved the best performance among four structures.

Graph neural network (GNN) uses graph theory to process data on the graph level (Scarselli et al., 2008). In order to construct a convolutional neural network (CNN) specifically tailored for graph-structured data, an approach employed a semi-supervised learning method. This method utilized a convolutional structure that operated by employing a localized first-order approximation of spectral graph convolutions (Kipf and Welling, 2016). EEG data is typically recorded from multiple electrodes, which can be treated as graph-structured data. Graph convolutions were introduced to process EEG data based on the topological structure of the electrodes, where edges between nodes in the graph were defined and weighted according to the geodesic distance. The resulting EEG- Graph Convolutional Neural Network (GCNN) showed a marked improvement in diagnosing neurological diseases (Wagh and Varatharajah, 2020). Li et al. (2021) proposed a patient-specific EEG seizure prediction model based on the Spatial–Temporal-Spectral Hierarchical Graph Convolutional Network Architecture (STS-HGCN-AL) framework. Two variant graph convolutions were produced to better capture the preictal EEG transitions in the hierarchical spatial–temporal-spectral level. The competitive results showed that the model could predict seizures efficiently.

As a rare disease, there are no public databases available for studying anti NMDA receptor encephalitis. Furthermore, there is a paucity of research that utilizes machine learning methods to classify EEG data for this condition. In this study, we collected EEG data from multiple patients with encephalitis as well as healthy controls. Based on this data, we proposed a model called TT-SGCN to classify EEG signals from different types of encephalitis and healthy controls.

## 2. Materials and methods

### 2.1. Participants

The study recruited participants from inpatient and outpatient children at the Department of Pediatrics, Second Hospital of Sichuan University between January 1, 2012, and October 31, 2021. Three groups of participants were included: anti NMDA receptor encephalitis group, viral encephalitis group, and health control group. The group with anti NMDA receptor encephalitis fulfilled the diagnostic criteria for this condition as outlined in the “Clinical Diagnostic Criteria for Autoimmune Encephalitis” published in the Lancet Neurology journal in 2016. On the other hand, the viral encephalitis group was diagnosed with a definitive etiology. The health control group in this study was not specifically recruited. Instead, they were selected among children who visited the hospital for neurological indications (e.g., suspected seizures), and their EEG data were recorded. A professional clinician reviewed the EEG recordings and found no evidence of abnormality. Table 1 shows the inclusion criteria for each group and the relevant characteristics of involved participants are presented in Table 2. The study was approved by the Ethics Committee of the West China Second University Hospital.

**Table 1:** Inclusion criteria for participants in three groups of anti NMDA receptor encephalitis, viral encephalitis, and health control.

Group of subjects	Inclusion criteria
Anti NMDA receptor encephalitis	The diagnostic criteria for anti NMDAR receptor encephalitis in the “Clinical Diagnostic Criteria for Autoimmune Encephalitis”(Graus et al., 2016) were met: six main clinical symptoms: (1) behavioral abnormalities (abnormal psychiatric behavior) and/or cognitive dysfunction; (2) language dysfunction manifested by reduced speech, silence, or incessant speech; (3) seizures; (4) movement disorders; (5) reduced level of consciousness; and (6) impaired autonomic function or central hyperventilation. Presence of one or more of the six major clinical symptoms with positive cerebrospinal fluid and/or serum anti-GluN1 antibody test, while excluding other diseases. Age under 18. At least 1 raw EEG data in the acute phase is obtained.
Viral encephalitis	There are clinical signs of brain parenchymal damage, and antibody IgM to a definitive virus, or viral nucleic acid, is detected in cerebrospinal fluid and/or serum. Cerebrospinal fluid results of laboratory tests meet international diagnostic criteria for viral encephalitis (Costa and Sato, 2020). Age under 18. At least 1 raw EEG data in the acute phase is obtained.
Health control	Children without severe neurological chronic diseases or acute illnesses examined in our EEG room. Age under 18. At least 1 raw EEG data in the acute phase is obtained.

**Table 2:** Characteristics of involved participants.

Characteristic	Anti NMDAR receptor encephalitis	Viral encephalitis	Health control
No. of participants	55	58	35
No. of males	22	36	18
Age (year)	8.1 ± 3.6	4.9 ± 3.8	5.6 ± 3.3

NIHON means No. of participants collected by NIHON KOHDEN and ANDY means No. of participants collected by Meren Andy.

### 2.2. EEG recording

This retrospective study spanned multiple years. The EEG equipment used in this study included two types: NIHON KOHDEN EEG-9200 K from Japan and Meren Andy AE-2010 from China. The electrodes were placed according to the international 10–20 system layout, with 18 typical electrodes (Fp1, Fp2, F3, F4, C3, C4, P3, P4, O1, O2, F7, F8, T3, T4, T5, T6, A1, A2) used for acquisitions. Different durations of EEG data in awake resting state were collected for multiple participants. A clinical specialist was responsible for the electrode layout and data acquisition to ensure the validity of the signal. In the current study, EEG data was acquired at awake resting state and the collection time for participants varied due to its retrospective nature; however, a minimum of 2 h of data collection was ensured. Considering that the subjects involved in the study were children, it was noticed that they tended to move around during the collection process. This movement often caused electrode detachment, which in turn led to excessive interference and motion artifacts in EEG signals. To mitigate this issue, a professional doctor thoroughly reviewed all the collected EEG data. The doctor utilized their expertise to identify and remove the signals that exhibited excessive interference, ultimately retaining only the relatively effective EEG data.

### 2.3. Proposed model

**Figure 1:**
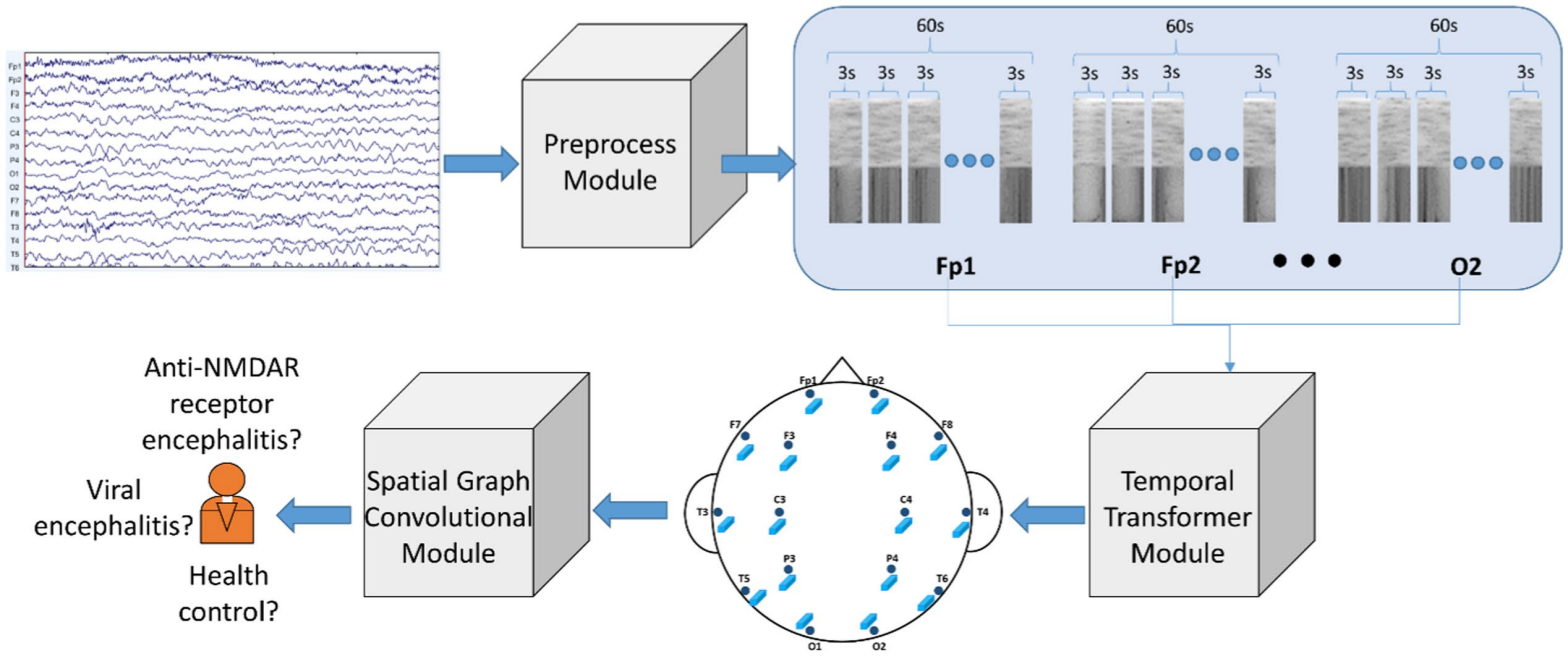
Architecture of proposed model: Temporal Transformer-Spatial Graph Convolutional Network (TT-SGCN). The original EEG signal underwent pre-processing steps such as filtering, z normalization, data segmentation, and STFT in the Preprocessed Module. Next, the Temporal Transformer Module utilized a transformer-based block to extract time-frequency information for each EEG channel. The features of all channels were then merged using graph convolutional methods in the Spatial Graph Convolutional Module, and the final classification results were obtained from this module.

We constructed a model called Temporal Transformer-Spatial Graph Convolutional Network (TT-SGCN), as shown in Figure 1 to explore the classification of EEG signal. The model consists of Preprocess Module, Temporal Transformer Module (TTM) and Spatial Graph Convolutional Module (SGCM).

### 2.4. Preprocess Module

EEGLAB (Delorme and Makeig, 2004) is a MATLAB-based toolkit for EEG data pre-processing. The EEG data collected during the awake resting state was combined for each participant, using the labeled awake marker assigned during EEG data recording. Subsequently, the data underwent processing through two filters, the first of which had a low frequency filtering of 0.1 Hz and a high frequency filtering of 70 Hz, while the second filter eliminated 50 Hz industrial frequency interference. The filtered data was subjected to Independent Component Analysis (ICA) to produce 21 components. These components were carefully examined by an experienced doctor to remove noise interference components. The reference signal for 16 electrodes (A1 and A2 excluded) was calculated by taking the average value of all electrodes as the baseline. Z-score normalization was then applied, as shown in the following formula:


(1) $$X^{*}=\frac{X-\mu }{\delta }$$

X represents the processed reference signal, where μ denotes its mean value and δ denotes its standard deviation. The EEG data for each participant was then divided into multiple segments, each containing 60 s of EEG data, which were further divided into twenty 3-s EEG data pieces. Short-time Fourier transform (STFT) was performed on each 3-s EEG data piece to produce a power density map. The twenty power density maps for each segment were concatenated in the time direction to form a concatenated power density (CPD) map. The CPD map for each EEG segment was used as input for the proposed model.

### 2.5. Temporal Transformer Module

**Figure 2:**
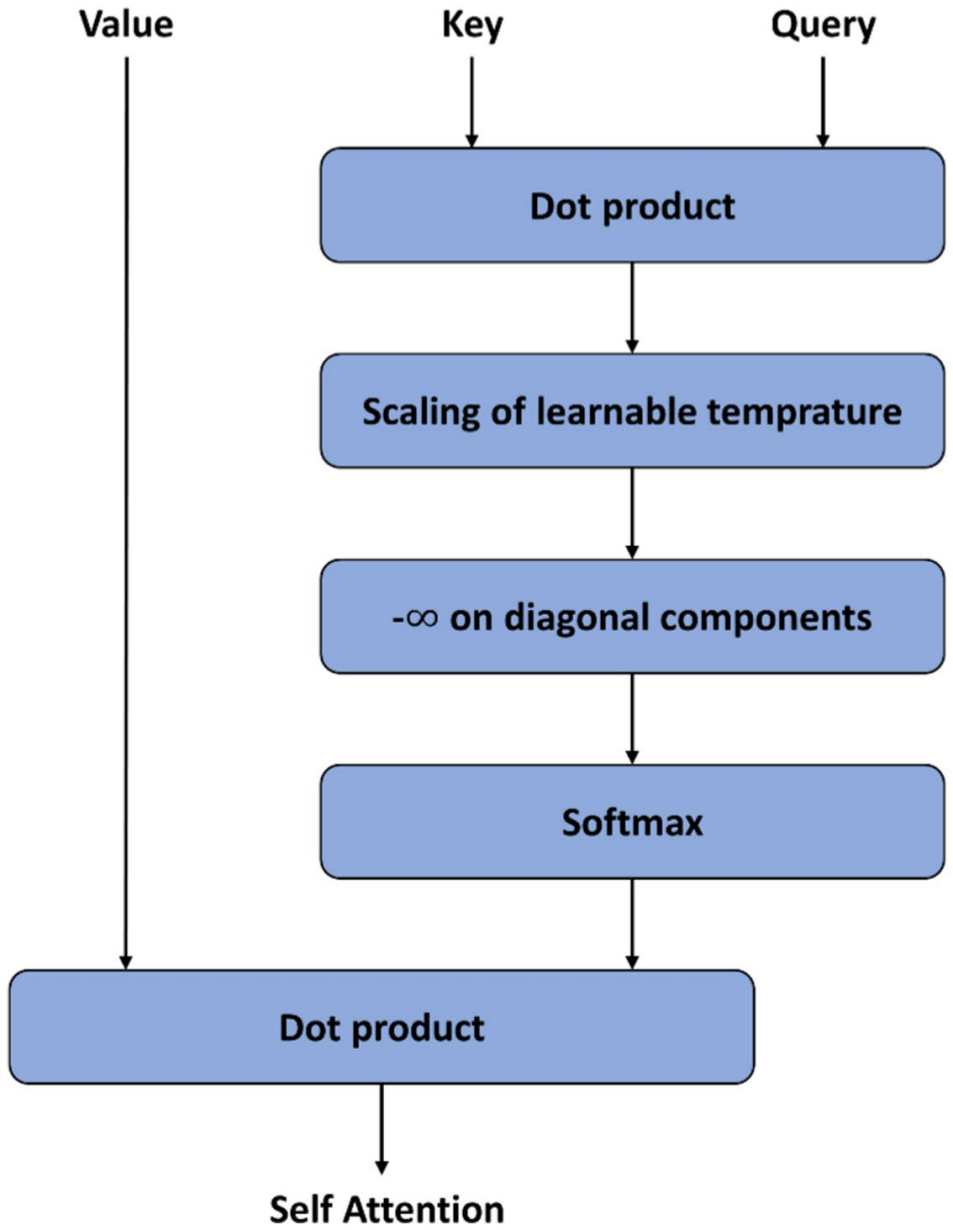
Structure of Locality Self-Attention (LSA) in Temporal Transformer Module (TTM).

To address the small-size dataset, we introduced a transformer-based module (Lee et al., 2021) to process the CPD maps generated from the EEG data. TTM introduced blocks of Shifted Patch Tokenization (SPT) and Locality Self-Attention (LSA) to enhance spatial information and increase locality inductive bias. Using the SPT technique, the input image was shifted in various directions and subsequently concatenated. The concatenated image was then divided into patches, following a similar approach to the standard Vision Transformer (ViT) methodology (Dosovitskiy et al., 2020). The basic block in transformer is Scaled Dot-Product Attention, which uses three different weight matrices to produce the queries vector (*Q*), key vector (*K*), and values vector (*V*), respectively. The calculation of Scaled Dot-Product Attention is shown in the following equation:


(2) $$Attention\left(Q,\;K,\;V\right)=softmax\left(\frac{QK^{T}}{\sqrt{d_{k}}}\right)V$$

which computed the dot product of *Q* and *K*, then divided by $\sqrt{d_{k}}$. $d_{k}\;$is the dimension of *K*. Then a softmax function was used to get the weights to multiply by *V*. The Multi-Head Attention consisted of multiple Scaled Dot-Product Attention layers. LSA firstly set -∞ on diagonal components of matrix, which was produced by dot product operation of Query and Key. As a result, the transformer block would prioritize attention other tokens other than its own, leading to a broader scope of attention. Secondly, LSA introduced learnable temperature scaling, which computes the softmax temperature during the learning process. This sharpens the distribution of attention scores. As a result, LSA also increases the locality inductive bias in general. The structure of LSA is shown in Figure 2.

### 2.6. Spatial Graph Convolutional Module

**Figure 3:**
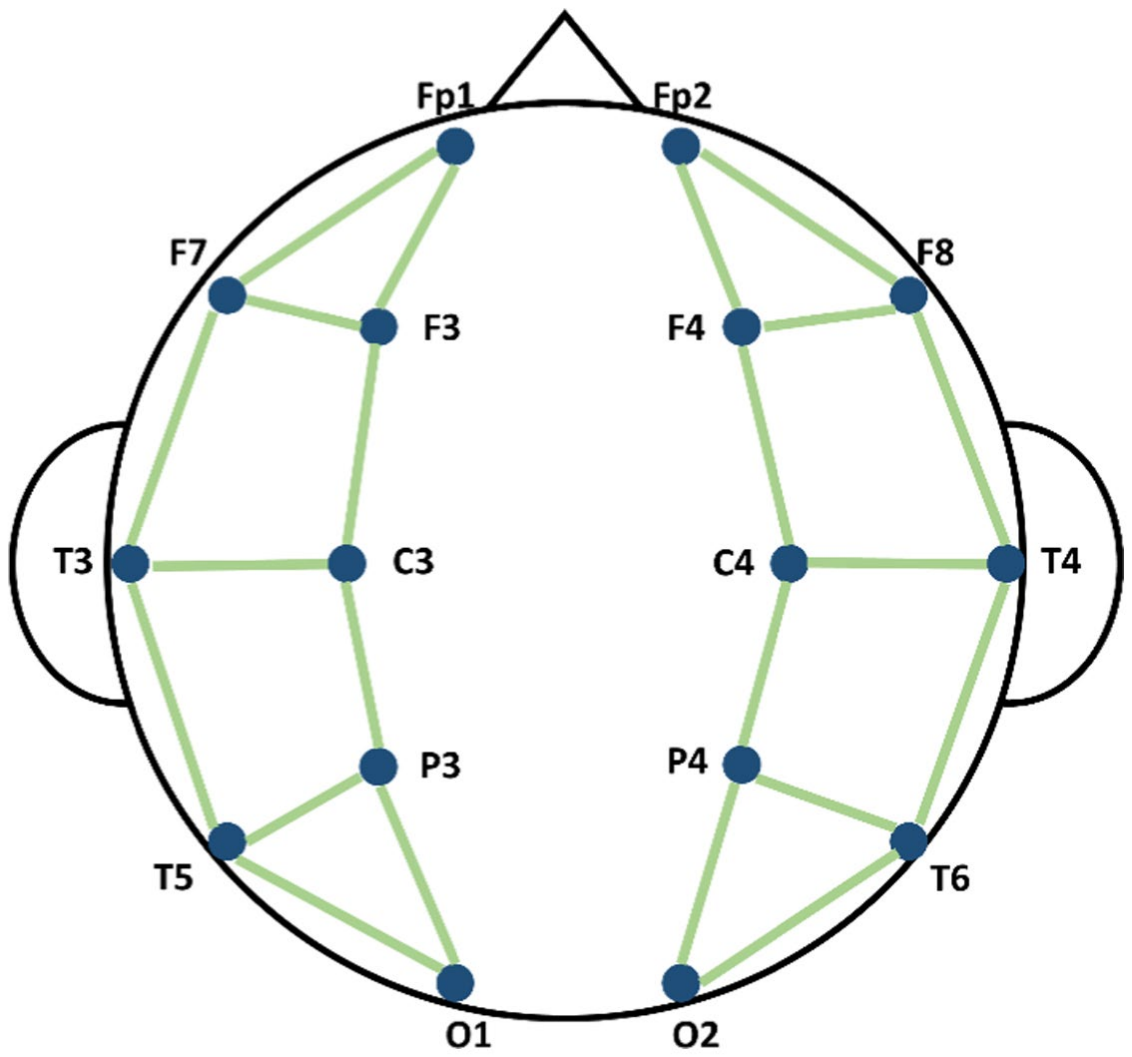
Schematic diagram of the connection methods of nodes in Spatial Graph Convolutional Module (SGCM).

We applied a graph network to introduce the topological structure of EEG electrodes. The graph was represented as G={V,E}, where V indicated the nodes of graph corresponding to the EEG electrodes, E indicated the edges connecting the nodes. The data of each node for the graph was represented by $X\in {\mathbb{R}}^{d\times 1}$, where d indicated the dimension per node. The edges linking the nodes were identified and represented using an adjacency matrix $A\in {\mathbb{R}}^{n\times n}$, where n indicated the number of nodes. The element $A_{ij}$ in the adjacency matrix was


(3) $$A_{ij}=\{\begin{array}{c} 1,for\;x_{i}⊕x_{j}\\ 0,for\;x_{i}⊘x_{j} \end{array}$$

where i and j were the indexes of nodes, ⊕ indicated that the connecting edge existed and ⊘ indicated that the connecting edge did not exist. Different researchers have used different possible connection modes between electrodes. Considering the distance between different nodes, we have defined the connection of nodes referring the conception on connecting nodes by previous study (Song et al., 2018; Zeng et al., 2020), as shown in Figure 3. The spectral graph convolution propagation rule was used based on the defined graph structure (Kipf and Welling, 2016). We built L number of graph convolutional layers and each layer produced its output feature according to the equation below:


(4) $$H^{\left(l+1\right)}=\sigma \left({\hat{D}}^{\frac{1}{2}}\hat{A}{\hat{D}}^{-\frac{1}{2}}H^{\left(l\right)}W^{\left(l\right)}\right)$$

where $\hat{A}=A+I_{N}$ is the adjacency matrix with added self-connections represented by the identity matrix. ${\hat{D}}_{ii}$ indicated the degree matrix of $\hat{A}$ with ${\hat{D}}_{ii}=\underset{j}{\sum }{\hat{A}}_{ij}$. $W^{\left(l\right)}$ was a trainable weight matrix corresponding to a specific layer. $\sigma \left(\cdot \right)$ was an activation function. $H^{\left(0\right)}=X$.

### 2.7. Training procedure

**Figure 4:**
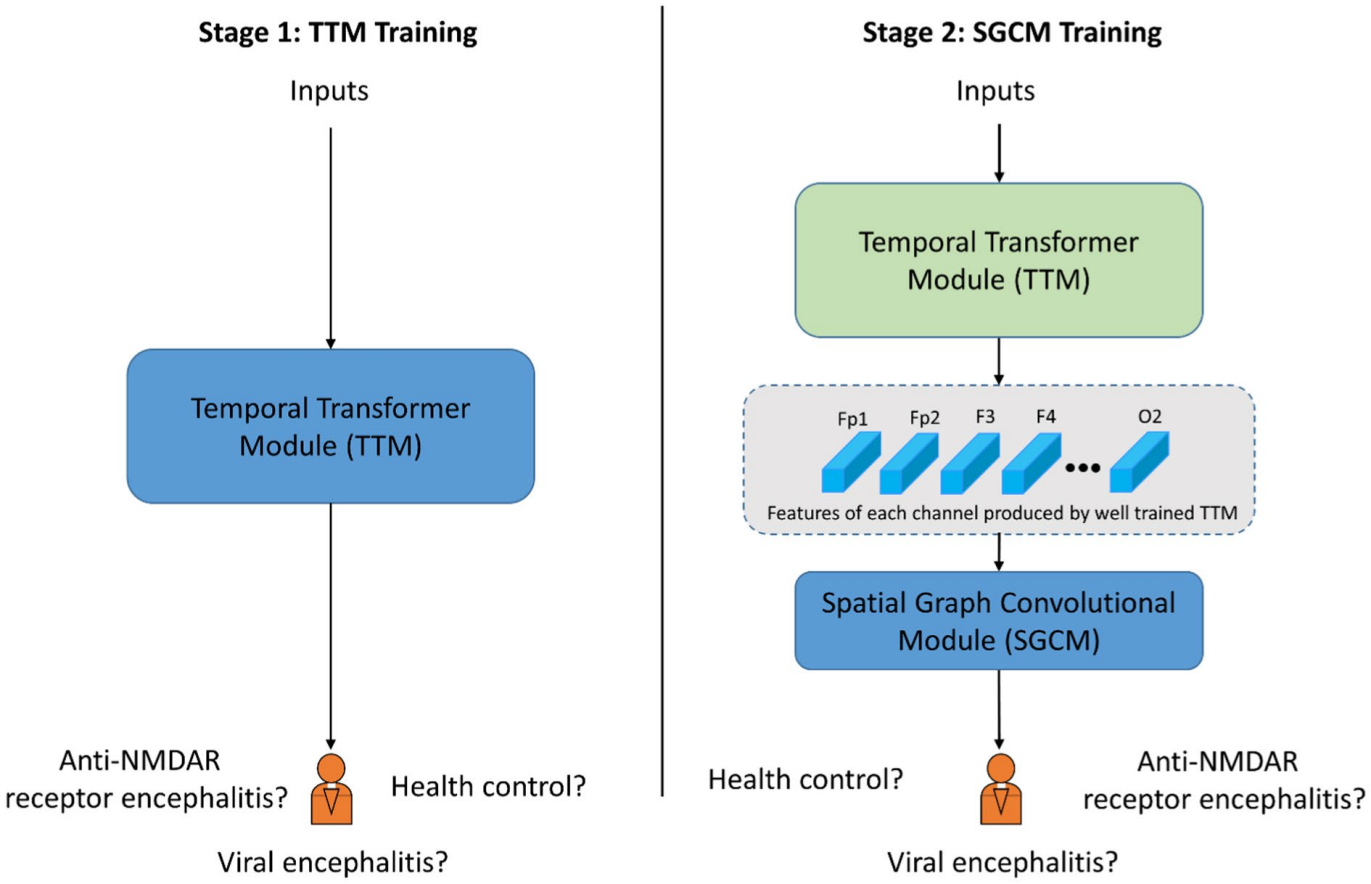
Schematic diagram of training procedure with two stages. The blue block indicates the corresponding module is under training. The green block indicated the corresponding module is already well trained and the parameters is fixed.

The training process consisted of two stages, as shown in Figure 4. In the stage 1, the TTM was applied to extract the dominant features from the time-frequency characteristics of each EEG channel based on the CPD map. The TTM was trained with epoch = 150, learning rate = 1e-4. In Stage 2, the GCM was employed to incorporate spatial characteristics of the EEG data by utilizing data collected from EEG electrodes. The GCM was trained with epoch = 80, learning rate = 1e-4. In both stages, ADAM optimizer (Zhang, 2018) was applied and used the cross entropy loss function (Zhang and Sabuncu, 2018).

The proposed model were evaluated using a 10-fold cross-validation strategy (Anguita et al., 2012). The dataset was divided into ten folds, with each fold used as the test set in turn and the remaining data used as the training set. The proposed model was trained on the training set and evaluated on the test set. The results were averaged over the ten test sets. The dataset consisted of a total of 1,599 samples, including 488 samples from the health control group, 434 samples from the anti NMDAR receptor encephalitis group, and 677 samples from the viral encephalitis group. Samples from the same subject were placed in the same training set or test set, so the performance of classification in this study was cross-subjects.

Firstly we conducted the classification of all three groups, followed by the three pairwise classifications among the three groups. In order to compare the performance of our proposed model with other popular methods for image processing, we have introduced the ResNet (He et al., 2016) and ViT (Dosovitskiy et al., 2020) networks. The performance of the classification was evaluated by four indicators (Chicco and Jurman, 2020; Powers, 2020): accuracy, recall, precision, and F1 score based on True-Positive (TP), True-Negative (TN), False-Positive (FP), and False-Negative (FN). The corresponding formulation are as follows:


(5) $$Accuracy=\frac{TP+TN}{TP+TN+FP+FN}$$

(6) $$\mathrm{Recall}=\frac{TP}{TP+FN}$$

(7) $$\mathrm{Precision}=\frac{TP}{TP+FP}$$

(8) $$F1=2\;\cdot \;\;\frac{Precision\cdot Recall}{Precision+Recall}$$

## 3. Results

### 3.1. Classification of all three groups

**Figure 5:**
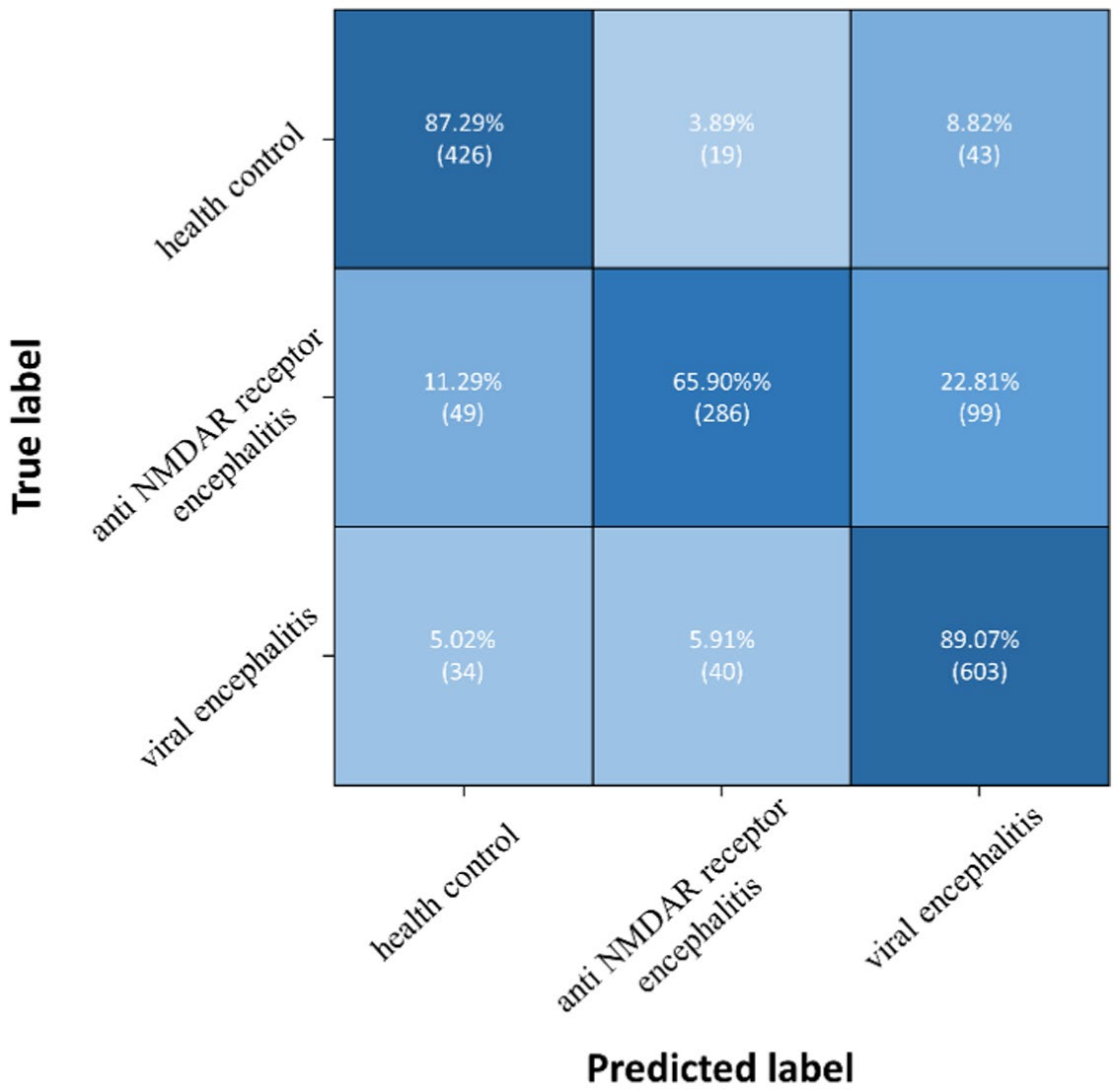
Confusion matrix of TT-SGCN after two stages of training in classification of all three groups.

**Figure 6:**
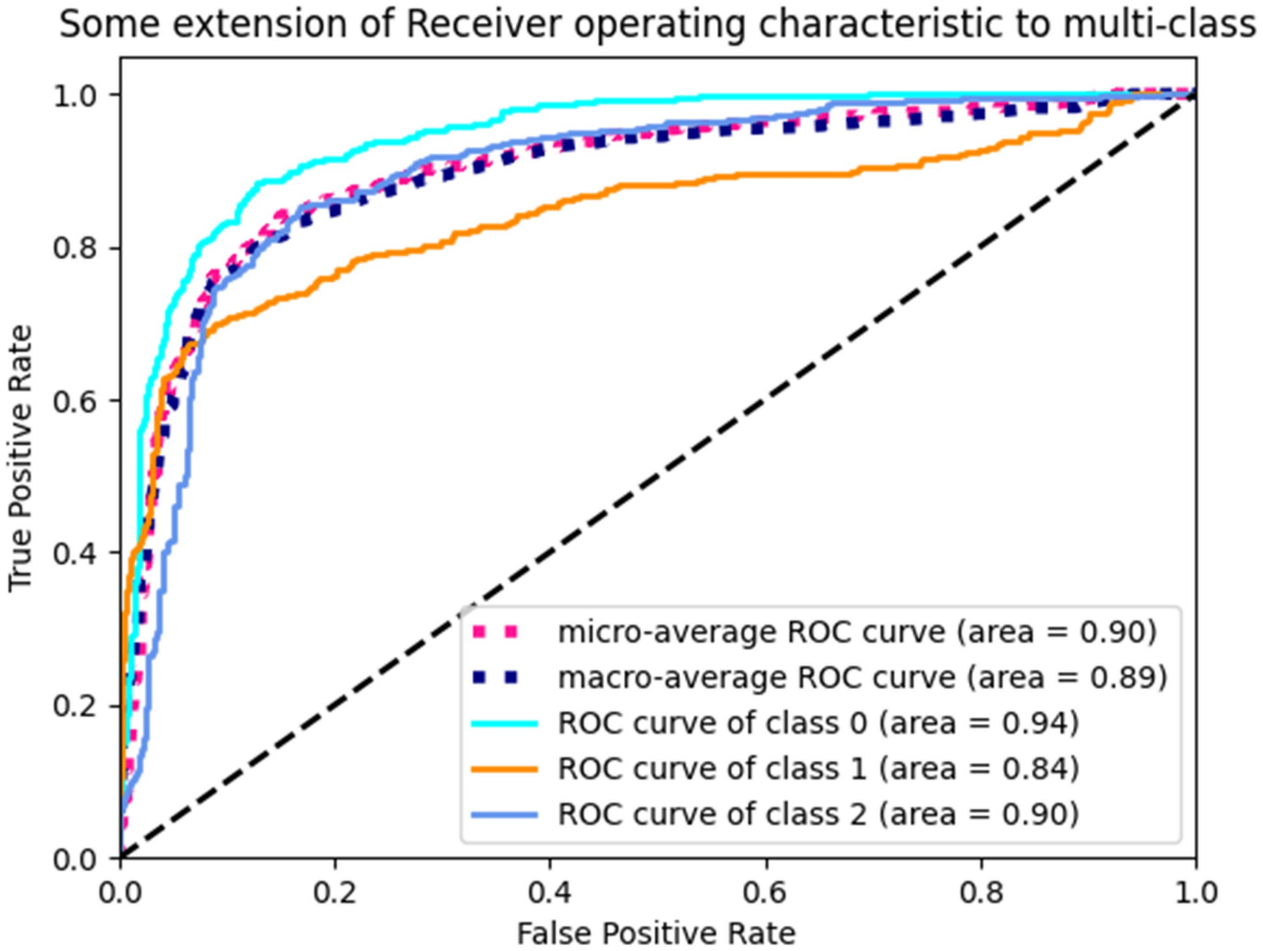
ROC curves of TT-SGCN after two stages of training in classification of all three groups. Class 0 indicates health control, class 1 indicates anti NMDAR receptor and class 2 indicates viral encephalitis.

The proposed TT-SGCN model was trained to classify EEG data from three groups: health control, anti NMDAR receptor encephalitis, and viral encephalitis. After the training stage 1, the TTM have achieved an accuracy of 67.03 to 77.08%, as shown in Table 3, in classifying each channel’s CPD map, indicating good performance. After the training stage 2, the confusion matrix in Figure 5 showed that our model could better identify EEG signals of health control and viral encephalitis, with accuracies of 87.29 and 89.07%, respectively. However, the identification of anti NMDAR receptor encephalitis was lower, with 22.81% of samples misclassified as viral encephalitis. The overall performance of the model in classifying the three groups was shown in Table 4. Compared to Resnet and ViT, our model achieved the best results with an accuracy of 82.23%, recall of 80.75%, precision of 82.51%, and F1 score of 81.23%. Additionally, the model’s classification results were visualized using the ROC curves in Figure 6, which showed good outcomes with the values of AUC.

**Table 3:** Accuracies of stage 1 for each channel using TTM in classification of all three groups.

Name of channel	Accuracy
Fp1	73.35%
Fp2	74.33%
F3	75.47%
F4	74.40%
C3	75.69%
C4	76.93%
P3	76.43%
P4	76.97%
O1	71.43%
O2	74.08%
F7	72.67%
F8	74.87%
T3	72.16%
T4	75.42%
T5	67.03%
T6	77.08%

**Table 4:** Performance of TT-SGCN after two stages of training in classification of all three groups.

Network	Accuracy	Recall	Precision	F1 score
Resnet	75.79%	74.38%	74.53%	74.37%
ViT	68.79%	66.03%	66.34%	65.72%
TT-SGCN(ours)	82.23%	80.75%	82.51%	81.23%

### 3.2. Pairwise classification among the three groups

**Figure 7:**
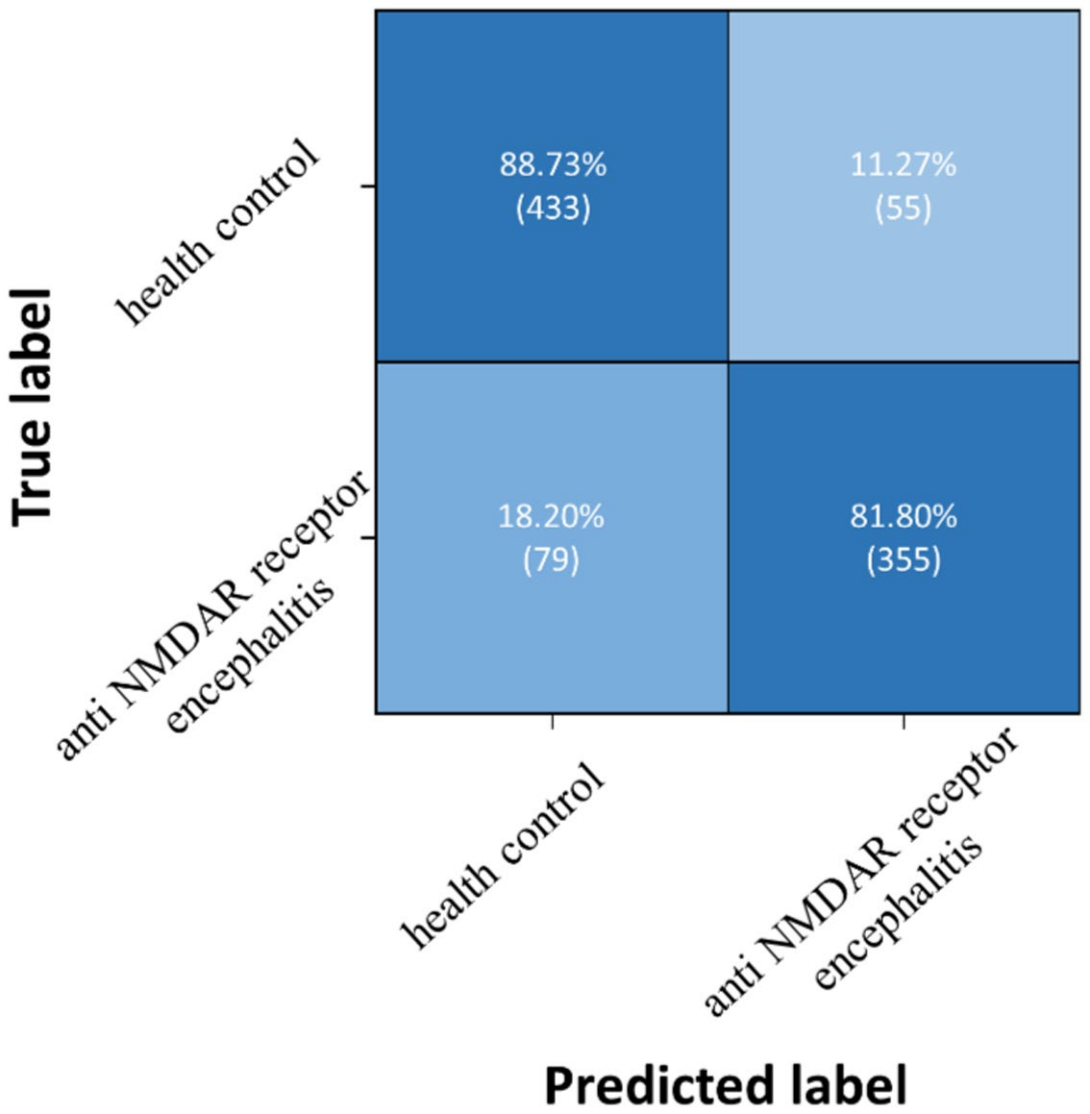
Confusion matrix of TT-SGCN after two stages of training in pairwise classification of health control and anti NMDA receptor encephalitis.

**Figure 8:**
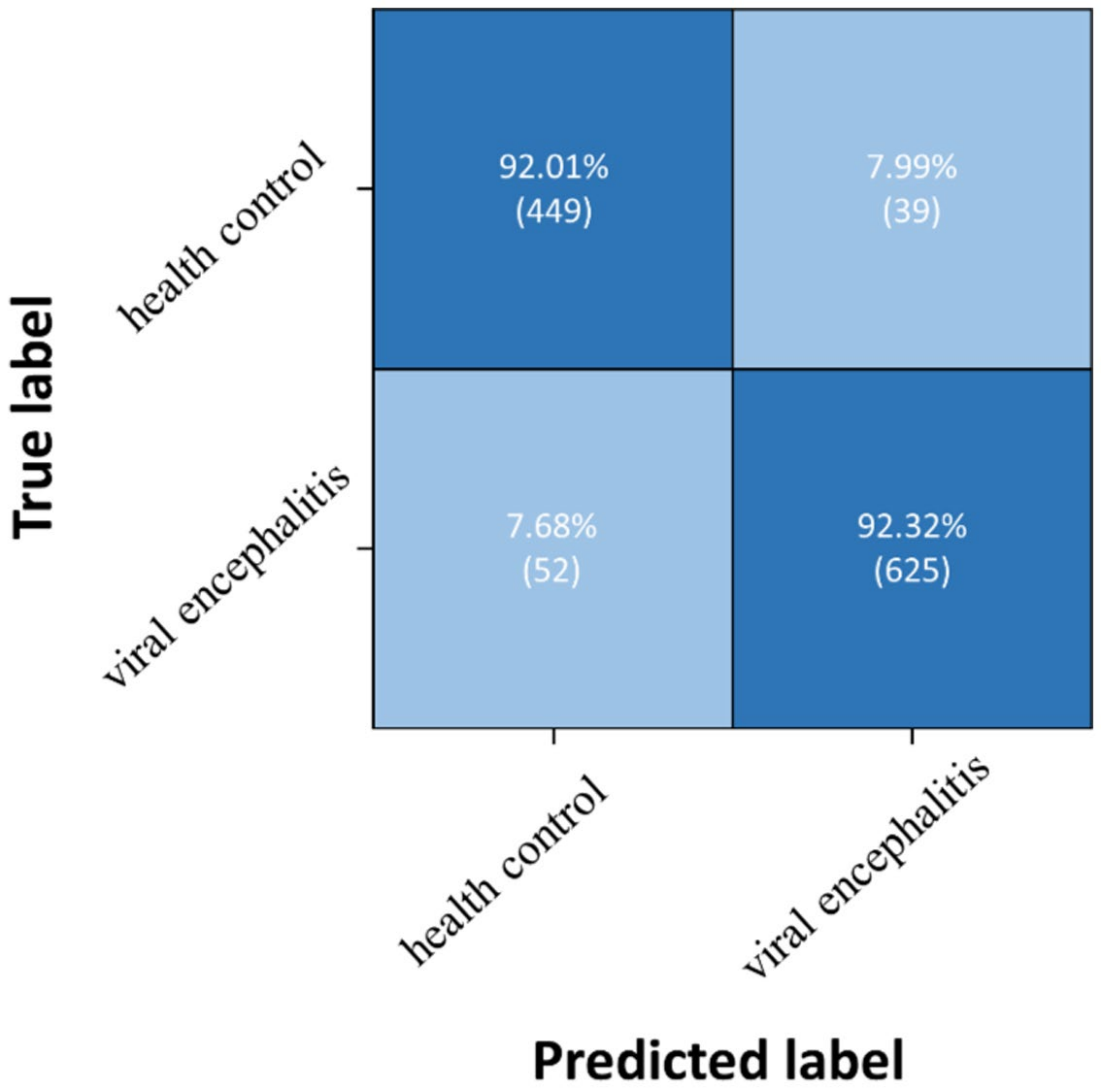
Confusion matrix of TT-SGCN after two stages of training in pairwise classification of health control and viral encephalitis.

**Figure 9:**
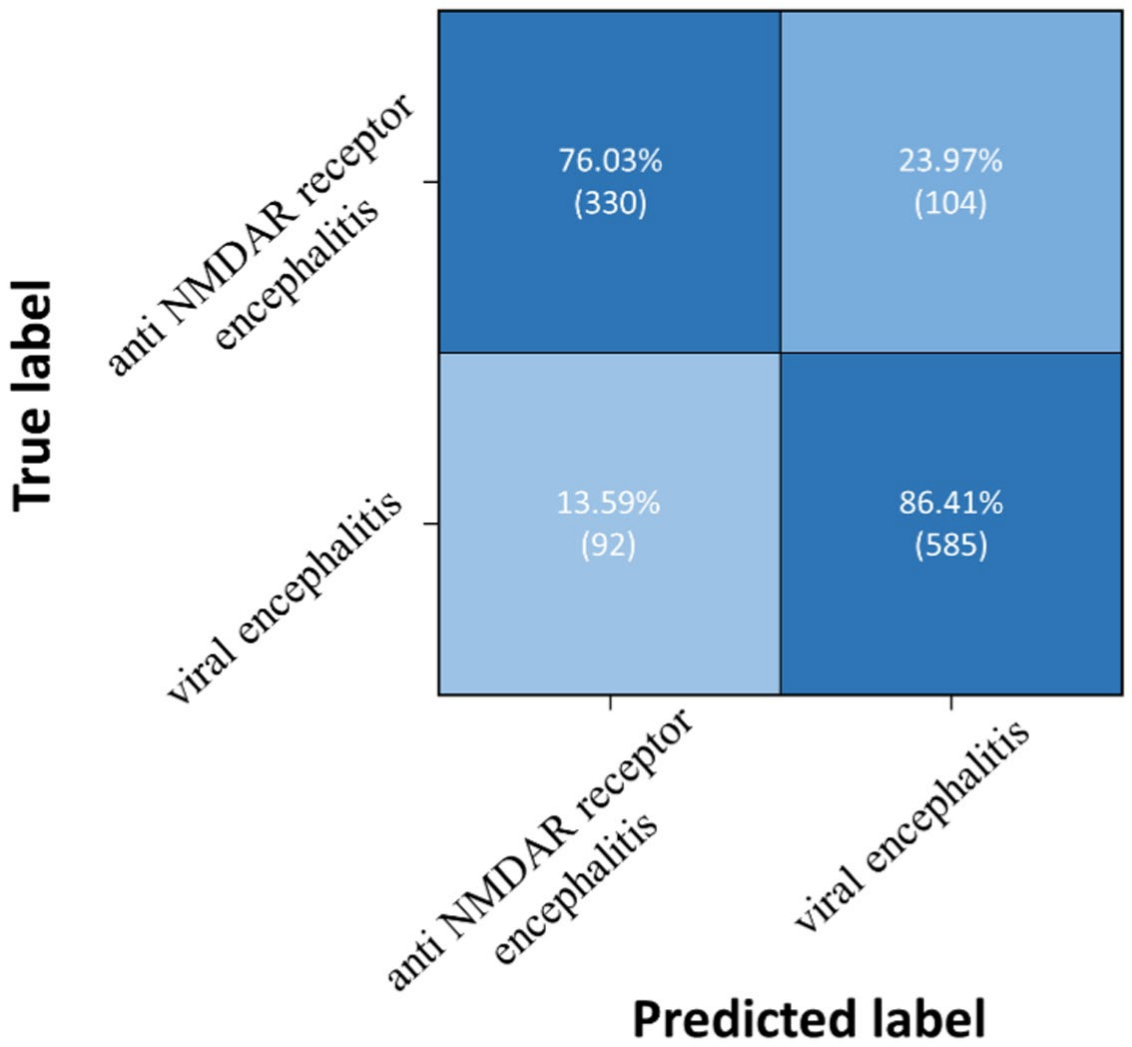
Confusion matrix of TT-SGCN after two stages of training in pairwise classification of anti NMDA receptor encephalitis and viral encephalitis.

Three pairwise classifications were performed, including health control-anti NMDAR receptor encephalitis, health control-viral encephalitis, and anti NMDAR receptor encephalitis-viral encephalitis. The results of classification after the second training stage were presented in Table 5. Confusion matrices were calculated for pairwise classification of health control and anti NMDAR receptor encephalitis in Figure 7, health control and viral encephalitis in Figure 8, and anti NMDAR receptor encephalitis and viral encephalitis in Figure 9. Overall, the proposed TT-SGCN model achieved good performance to distinguish between the health control group and encephalitis control group. Additionally, TT-SGCN achieved the highest level of performance in distinguishing between the healthy control group and viral encephalitis control group, with an accuracy of 92.18%, recall of 92.01%, precision of 89.62%, and F1 score of 90.79%. The pairwise classification performance of anti NMDAR receptor encephalitis-viral encephalitis among the three groups was the lowest; however, our model still showed the best results.

**Table 5:** Performance of TT-SGCN after two stages of training in pairwise classification among three groups.

Network	Accuracy	Recall	Precision	F1 score
Health control—anti NMDA receptor encephalitis				
Resnet	81.34%	84.22%	81.22%	82.69%
ViT	80.47%	85.45%	79.27%	82.24%
TT-SGCN(ours)	85.46%	88.72%	84.57%	86.60%
Health control—viral encephalitis control				
Resnet	87.98%	83.60%	87.17%	85.35%
ViT	88.75%	85.24%	87.57%	86.39%
TT-SGCN(ours)	92.18%	92.01%	89.62%	90.79%
Anti NMDA receptor encephalitis—viral encephalitis control				
Resnet	79.92%	65.66%	79.38%	71.87%
ViT	78.21%	76.95%	70.16%	73.40%
TT-SGCN(ours)	82.35%	76.03%	78.19%	77.10%

## 4. Discussion

The power density map of EEG data provides important time and frequency domain information, which is a crucial feature for distinguishing different types of EEG signals. Previous researchers have identified the EEG representation of specific encephalitis subtypes (Gable et al., 2009; Schmitt et al., 2012; Gitiaux et al., 2013; Huang et al., 2016; Zhang et al., 2017; Gillinder et al., 2019). In our analysis we compared two encephalitis subtypes (viral and anti-NMDAR encephalitis) with healthy controls, using a two stage approach. In the first stage we used TTM to extract time-frequency information using a transformer-based block and this showed reasonable classification accuracies for each channel separately, as shown in Table 3. Moreover, the EEG signals from C3, C4, P3, P4, and T6 showed the highest accuracies, indicating significant differences in EEG characteristics in the corresponding brain regions of the parietal lobe, central lobe, and right temporal lobe. This suggests that clinicians may be able to efficiently identify different encephalitis with EEG representation from specific EEG channels.

The proposed model, employing the SGCM, demonstrated enhanced classification performance for all three groups using features from 16 recording channels generated by TTM. The EEG curves for healthy participants and participants with encephalitis exhibited significant differences, resulting in better classification of the EEG data for the health control and encephalitis groups. Although distinguishing between the EEG curves of anti NMDAR receptor encephalitis and viral encephalitis proved challenging, a clinical specialist reviewed the recordings and observed normal posterior head rhythm, focal slow waves, extreme β activity, and focal slow waves in the frontal and frontal lobe regions in the anti NMDAR receptor encephalitis group and diffuse slow waves and abnormal interictal discharges in the viral encephalitis group. It is not known which of these observed differences may have contributed most to the classification performance of the models. Future work is needed to make the performance of such models more clinically interpretable.

According to observations by a clinical specialist, normal posterior head rhythm, focal slow waves, extreme β activity, and focal slow waves in the frontal and frontal lobe regions are commonly observed in anti NMDAR receptor encephalitis. Conversely, diffuse slow waves and abnormal interictal discharges are frequently found in viral encephalitis. These characteristics may contribute to identifying anti NMDAR receptor encephalitis.

We conducted two types of experiments using our proposed model, TT-SGCN: classification of all three groups and pairwise classification. In both experiments, our model outperformed Resnet and ViT, with better results in terms of accuracy, recall, precision, and F1 score. In comparison to classification modeling with large natural image datasets, the data utilized in this experiment is of small size. By employing the transformer-based structure specifically designed for small-scale image datasets (Lee et al., 2021), TTM demonstrated remarkable feature extraction capability, leading to the attainment of competent classification accuracy based on the CPD map of each channel. Taking into account the location of EEG recording nodes, the utilization of SGCM effectively enhanced accuracy and yielded improved values compared to the single stage (TTM) approach for recall, precision, and F1 score in classifying the EEG signal of all three groups. This was accomplished by merging the features of each EEG electrode using the graph convolutional method (Kipf and Welling, 2016). Since a well-trained model to classify fewer categories may be more efficient in extracting key features, specific encephalitis was easier to identify with higher metrics in the pairwise classification experiment. However, a well-trained model capable of classifying all three groups is more practical for developing an intelligent diagnostic auxiliary tool for encephalitis.

## 5. Conclusion

We were pioneers in using machine learning methods to intelligently classify EEG data for encephalitis. Our proposed model, TT-SGCN, leverages the specific EEG representation of anti NMDAR receptor encephalitis to accurately classify the EEG signal of encephalitis. By using a transformer-based block and graph network method, our model achieved good performance in the classification of three groups, with an accuracy of 82.23%, recall of 80.75%, precision of 82.51%, and F1 score of 81.23%. This study offers novel insights for future research endeavors. By enlarging the sample size, it becomes feasible to construct expansive open datasets that can be utilized by other researchers. This facilitates greater collaboration and promotes further investigations in the field.

## 6. Limitations

In this study, despite extensive collection of clinical EEG data over an extended period, the constructed dataset remains limited in size. The effectiveness of neural network models is often dependent on the utilization of large-scale datasets, resulting in improved outcomes. To address this limitation, firstly, future initiatives could involve collaborative efforts with multiple sites to collect diverse EEG data pertaining to various encephalitis diseases. Subsequently, a large and specialized database can be established, facilitating accessibility for other researchers. Secondly, it is worth noting that the study employed two distinct types of EEG collection devices, potentially introducing heterogeneity in the collected signals. To enhance the comprehensiveness of the dataset, further exploration can be conducted, entailing a detailed analysis of EEG signals derived from different devices. In addition, a pre-set connection method was applied to employ the graph convolution method for electrode signals. Subsequent research endeavors should consider exploring the influence of node connection methods in diverse graph networks and investigating the correlations between hemispheres. Relevant research has barely been found concerning the quantitative classification results of EEG signals associated with encephalitis. Consequently, this study has focused solely on a comparison of various classic neural network models using the collected EEG data for classification purposes. It is recommended that future researchers refer to this article’s results or seek out the latest findings from other scholars for comparative analysis and further advancements in this field.

## Data availability statement

The raw data supporting the conclusions of this article will be made available by the authors, without undue reservation.

## Ethics statement

The studies involving humans were approved by Ethics Committee of the West China Second University Hospital. The studies were conducted in accordance with the local legislation and institutional requirements. Written informed consent for participation was not required from the participants or the participants’ legal guardians/next of kin because All guardians of the participants were orally informed about their involvement in this study.

## Author contributions

RD conceived the study and drafted the manuscript. RD and TY designed the experiments. TY collected the data. RD and YW analyzed the data. QW, RL, BH, and ZP edited the manuscript. All authors contributed to the article and approved the submitted version.

## Funding

This work was supported by “From 0 to 1” Original Innovation Project of the Basic Frontier Scientific Research Program of the Chinese Academy of Sciences (29J20-015-III), Key Laboratory of Spectral Imaging Technology, Xi’an Institute of Optics and Precision Mechanics of the Chinese Academy of Sciences, the Open Research Fund for development of high-end scientific instruments and core components of the Center for Shared Technologies and Facilities, XIOPM, CAS, the Key Laboratory of Biomedical Spectroscopy of Xi’an (39J18-136). Visual Interactive Control System in Intelligent Cockpit (1IJ23-026-II).

## Conflict of interest

The authors declare that the research was conducted in the absence of any commercial or financial relationships that could be construed as a potential conflict of interest.

## Publisher’s note

All claims expressed in this article are solely those of the authors and do not necessarily represent those of their affiliated organizations, or those of the publisher, the editors and the reviewers. Any product that may be evaluated in this article, or claim that may be made by its manufacturer, is not guaranteed or endorsed by the publisher.
